# Dating the period when intensive anthropogenic activity began to influence the Sanjiang Plain, Northeast China

**DOI:** 10.1038/srep22153

**Published:** 2016-02-24

**Authors:** Jinxin Cong, Chuanyu Gao, Yan Zhang, Shaoqing Zhang, Jiabao He, Guoping Wang

**Affiliations:** 1 Key Laboratory of Wetland Ecology and Environment, Northeast Institute of Geography and Agroecology, Chinese Academy of Sciences, 130102, Changchun, China; 2 ILÖK, Hydrology Group, University of Münster, 48149, Münster, Germany

## Abstract

Dating the start of intensive anthropogenic influence on ecosystems is important for identifying the conditions necessary for ecosystem recovery. However, few studies have focused on determining when anthropogenic influences on wetland began through sedimentary archives. To fill this critical gap in our knowledge, combustion sources and emission intensities, reconstructed via black carbon (BC) and polycyclic aromatic hydrocarbons (PAHs) were analyzed in two wetlands in the Sanjiang Plain in Northeast China. ^14^C provided age control for the sedimentary records. By combining previous sedimentary and archaeological studies, we attempt to date the beginning of intensive anthropogenic influences on the Sanjiang Plain. Our results showed that BC deposition fluxes increased from 0.02 to 0.7 g C/m^2^.yr during the last 10,000 years. An upward trend was apparent during the last 500 years. Before 1200 cal yr BP, human activities were minor, such that the wetland ecosystem in the Sanjiang Plain before this period may represent the reference conditions that for the recovery of these wetlands. As the human population increased after 1200 cal yr BP, combustion sources changed and residential areas became a major source of BC and PAHs. In this way, the wetland ecosystem gradually became more heavily influenced by human activities.

The historical conditions of an ecosystem that have been influenced by subsequent human activities—and the remaining information associated with these ecosystems—can be used to inform modern ecosystem management and restoration approaches^1^. Paleoenvironmental records can be used to reconstruct and understand the condition of these ecosystems (e.g., aquatic systems) in the past and assess the influence of climate changes and human activities on these ecosystems^2^,^3^. ‘Baseline’ conditions for ecosystem conservation and restoration are those conditions that were expected to characterize these ecosystems when human impacts were minimal^3^. Paleoenvironmental records provide a useful tool for identifying baseline conditions for ecosystem recovery. Identifying the period when human activities began to influence the ecosystem is the first step in determining the baseline conditions that are necessary for ecosystem restoration. The Sanjiang Plain, located in Northeast China, has records of human activity dating to the early Holocene epoch^4^. Human population in this area increased (Heilongjiang Province) from 20,000 in 8000 cal yr BP to 1.27 million in AD 1897 and to nearly 38.34 million in AD 2011^4^,^5^,^6^. As the human population has grown, the influence of human activities on wetland ecosystems on the Sanjiang Plain has undoubtedly increased, presumably increasing the extent of wetland ecosystems that have been destroyed^7^. Thus, identifying the period when human activities began to influence wetland ecosystems of the Sanjiang Plain and reconstructing these baseline conditions using paleoenvironmental records is critical.

Black carbon (BC), which is produced by the incomplete combustion of fossil fuels or biomass, is widespread in the environment and influences biogeochemical processes in ecosystems^8^. Previous studies have estimated that global BC emitted by vegetation fires ranges between 50 and 270 Tg/yr^9^. Fossil fuel combustion emitted 4.4 Tg/yr in AD 2000 around the world and has increased linearly in recent years^10^. As an important component of atmospheric aerosols, BC has an impact on global climate change^11^ and on the transport of persistent organic pollutants (POPs)^12^,^13^. After being retained in the atmosphere for a few days^14^, BC is deposited in the landscape and can be stored in the soil carbon pool for several thousand years^15^.

Previous studies have focused on investigating BC concentrations and historical deposition fluxes in forest soils^16^, marine sediments^17^, lake sediments^18^, and loess^19^. These studies have suggested that the historical trend of BC deposition fluxes is related to climate change^20^ or the degree of BC produced by anthropogenic sources^21^. This approach therefore is suitable for reconstructing the historical intensity of combustion source emissions. However, few studies of this type have focused on wetland ecosystems, which cover 5 to 8% of the Earth’s land surface and serve as key paleoenvironmental archives^22^. Thus, investigating historical fluctuations in BC deposition in wetland systems and the factors that influence these fluxes is critical. In addition, the climate of the Sanjiang Plain has changed dramatically during the Holocene epoch. The difference between the maximum and minimum temperatures in the Sanjiang Plain was nearly 6 °C^23^. This climatic variability has likely affected the frequency and intensity of wildfires^24^ and therefore may have influenced deposition fluxes of BC. Analyses of BC in wetland sediments can therefore be used to study the patterns and drivers of past combustion intensive (i.e., anthropogenic nature emission intensity) on the Sanjiang Plain, Northeast China.

Polycyclic aromatic hydrocarbons (PAHs) are organic pollutants prevalent in the sediments of freshwater environments^25^. PAHs are co-emitted with BC and are produced by similar historical combustion sources^26^. Higher concentrations of PAHs with low aromaticity, such as phenanthrene (PHE), anthracene (ANT), fluoranthene (FLT) and pyrene (PYR), are frequently related to combustion processes^27^. This relationship is a useful tool for diagnosing the sources of PAHs^28^. Once in the water-sediment system, PAHs can bind to suspended particulate matter and can be easily transported to surface sediments^29^. Based on the diagnostic ratios of PAHs in sedimentary archives, the sources of these combustion products can be identified, and the degree of influence of human activities on ecosystems can be evaluated. In addition to identifying modern combustion sources, historical sources of PAHs have already been reconstructed in sedimentary environments successfully^30^. The ratio of ANT/(ANT + PHE) and FLT/(FLT + PYR) produced by combustion sources were mostly higher than 0.1 and 0.5, respectively^27^,^31^. Therefore, these diagnostic ratios are suitable for investigating the historical types of combustion sources and can serve as indirect indicators for identifying the historical sources of combustion in wetland sediments.

This study presents the BC and PAH data from two sedimentary profiles in the Sanjiang Plain, Northeast China. We used these data to reconstruct variation in combustion emissions by BC deposition fluxes and diagnose historical types of combustion sources using the diagnostic ratios of PAHs during the Holocene epoch. Based on these results, we assessed the degree to which historical human activities have influenced BC and PAH deposition in wetland ecosystems of the Sanjiang Plain. By combining our data with previous sedimentary studies from this region and historical documents from the Heilongjiang Province, we aimed to determine the period when human activities began to influence wetland ecosystems in the Sanjiang Plain. The identification of this period will allow the characterization of the baseline conditions necessary for future wetland conservation practices.

## Results

### Physico-chemical characteristics of the profiles

**Figure 1:**
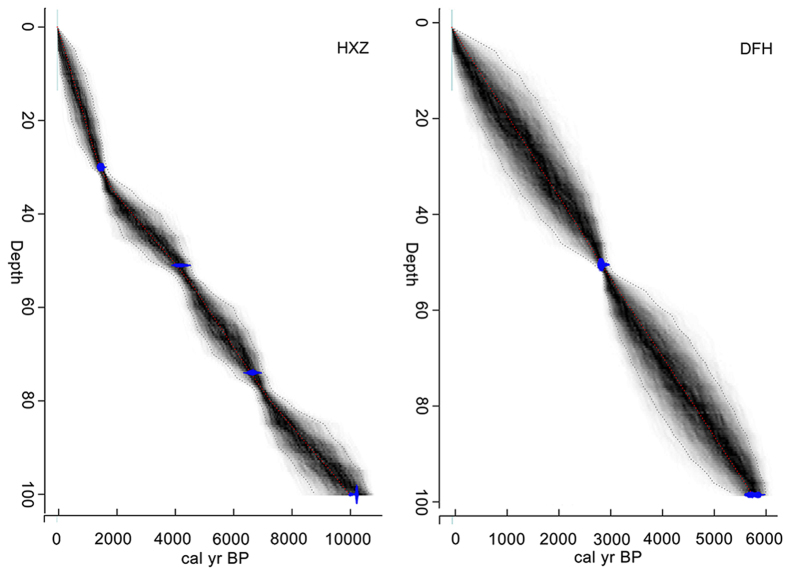
Age-depth model for the DFH and HXZ profiles. Gray scaling indicates all likely age-depth models, and the blue region indicates actual dates with 2-sigma error.

The ‘Bacon’ model in the R environment provided an estimation of the age-depth relationship with 95% confidence intervals and no outliers. The HXZ and DFH records date to 9900 cal yr BP and 5700 cal yr BP, respectively (Fig. 1). To compare the HXZ wetland with the DFH wetland, which only extends to 5000 cal yr BP, we chose the last 10,000 years to examine the historical trend in black carbon and the late Holocene epoch (last 4500 years) for determining the types of combustion sources in this study. The sedimentary rates obtained by the age-depth model were similar in different sections of the two profiles: the mean values were 0.10 mm/yr and 0.17 mm/yr in the HXZ and DFH profiles, respectively. Mean accumulation rate in the DFH profile was 2.07 g/m^2^.yr, twice as high as that of the HXZ profile (0.93 g/m^2^.yr). In the DFH profile, the dry bulk density in surface sediments was 0.38 g/cm^3^. This value increased with increasing depth, reaching a stable value of approximately 1.6 g/cm^3^ when the depth exceeded 40 cm. The dry bulk density in the HXZ profile increased from 0.31 to 0.90 g/cm^3^ in the top 50 cm.

### Concentrations of BC and PAHs in the two profiles

**Figure 2:**
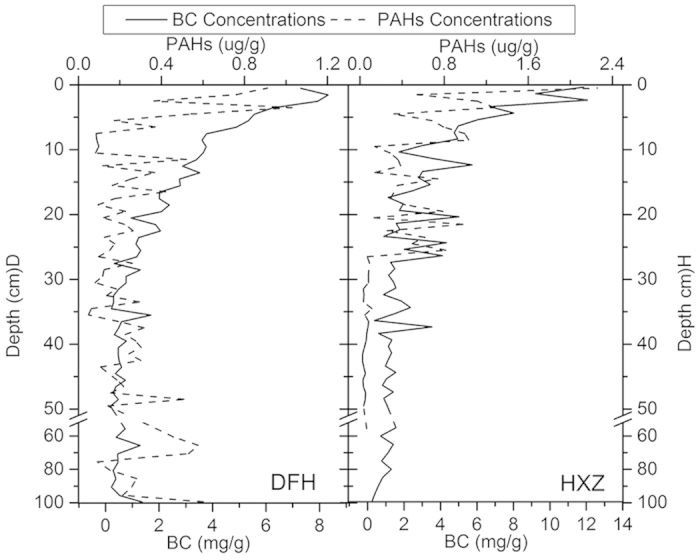
Variation in BC concentrations (mg/g) and PAHs concentrations (ug/g) with the profile depth in DFH and HXZ.

The concentrations of BC and PAHs in the two profiles with depth are shown in Fig. 2. In the DFH profile, BC concentrations ranged from 0.2 mg C/g to 8.3 mg C/g, with a mean of 1.8 mg C/g. These values were lower than those in the HXZ profile (0.3–12.1 mg C/g). The trend of BC with depth in the two profiles is similar: BC concentrations in top soils were higher than BC concentrations in bottom soils. As depth increased, BC concentrations in the two profiles decreased. One-way ANOVA revealed the BC concentrations in the two profiles were significantly different (P < 0.05). The trends in BC in the two profiles were similar in that the layers with high BC concentrations were primarily located at the surface. The concentrations of BC from locations in the top 20 cm declined significantly as depth increased in the two profiles. The BC concentrations in bottom sediments (beneath 20 cm) changed slightly as depth increased.

The trend for the eight PAH concentrations of the two profiles was similar to that for BC concentrations: the concentrations at the surface were significantly higher than concentrations at the bottom. One-way ANOVA revealed that there was no significant difference in PAH concentrations of the two profiles. Mean PAH concentration was 0.27 μg/g in DFH, which was slightly lower than in HXZ (0.36 μg/g). The ratio of the maximum total eight PAH concentrations and the minimum of the two profiles were 25 (DFH) and 113 (HXZ).

## Discussion

### Historical combustion intensities revealed by BC deposition fluxes

**Figure 3:**
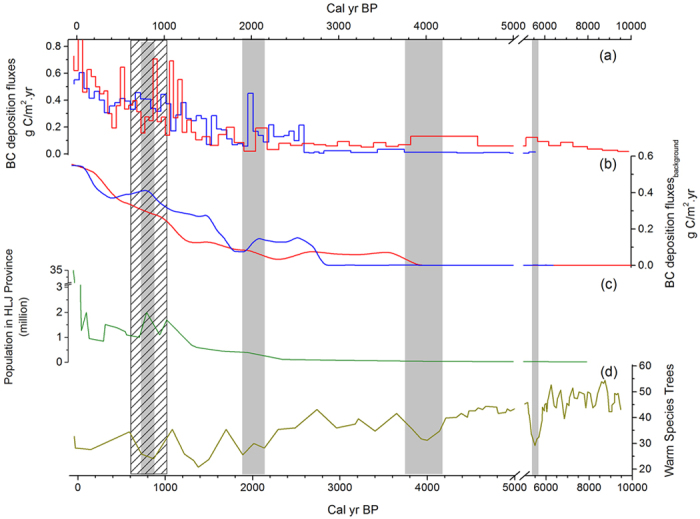
(**a**) Historical black carbon deposition fluxes in the DFH (blue line) and HXZ (red line) profiles during the Holocene epoch; (**b**) background of BC deposition fluxes in the two profiles obtained by Charanalysis1.1; (**c**) historical population of Heilongjiang (HLJ) Province during the Holocene epoch^4^,^5^,^6^; and (**d**) change in historical warm-species trees in Jingbo Lake (N 43.9°, E 128.7°) revealed by pollen analysis^34^.

The mean deposition fluxes of BC were 0.23 g C/m^2^.yr in the DFH profile and 0.25 g C/m^2^.yr in the HXZ profile. Maximum BC fluxes in the HXZ profile (0.91 g C/m^2^.yr) were higher than that in the DFH profile (0.60 g C/m^2^.yr). Fluxes of BC in the two profiles were not significantly different (P > 0.05), which may indicate that the historical sources of BC were similar for the different sites. Changing in BC deposition fluxes primarily appeared in the late Holocene epoch in the two profiles (Fig. 3a). BC fluxes increased in the two profiles, while the increasing rates changed for different periods during the Holocene. In the HXZ profile, the trend in BC fluxes was nearly flat before 1300 cal yr BP, and no clear increasing trend was observed during this period. However, the trend in BC fluxes increased from 0.1 g C/m^2^.yr to 0.9 g C/m^2^.yr after 1300 cal yr BP. A similar trend was observed in the DFH profile, where the boundary of the two periods was approximately 1500 cal yr BP. However, there existed a special period with higher BC deposition fluxes at approximately 2000 cal yr BP in the DFH profile.

Black carbon has primarily been produced by natural sources (e.g., surface plant incomplete combustion)^32^ and anthropogenic sources, such as residential burning^33^. Historical population characteristics of Heilongjiang (HLJ) province are shown in Fig. 3c. The earliest population records in HLJ Province were from approximately 8000 cal yr BP. During that period, humans had not yet been civilized, and few people (~20,000) were living there^4^. The population size gradually increased after 2000 cal yr BP. After the Tang Dynasty culture affected northern China (AD 682, 1268 yr BP), rates of population growth increased, and residents of Northeast China gradually began to farm. Owing to several wars (e.g., AD 726, 925, and 1114) in HLJ Province and changing of ethnic polices^4^, the population of HLJ Province fluctuated violently. After AD 1600, the population of HLJ Province continued to increase, especially in the last century: the population increased from 1.27 million in 1897 to 38.34 million in AD 2011^4^,^5^,^6^. Jingbo Lake (N 43.9°, E 128.7°), located in the western Sanjiang Plain, has similar climate characteristics to the study area during the Holocene epoch. Pollen analysis showed that there were four periods (4200-3800, 2200-1800, 800 and 600 cal yr BP) with cool climate during the last 5000 years^34^ (Fig. 3d). During these periods, background BC fluxes decreased, as shown by the HXZ profile. The degree of BC deposition fluxes decreased when the climate became cooler. The reason for this decrease may be the decline in the frequency and intensity of wildfires with low temperature^35^,^36^. However, variation in BC fluxes during the late Holocene epoch cannot be explained by climatic fluctuations. Because the climate during the late Holocene epoch alternated between cool and warm periods^34^, BC fluxes increased in both of the two profiles, especially during the last 1200 years. Before 1200 cal yr BP, the BC fluxes in two profiles were lower than 0.3 g C/m^2^.yr and the climate fluctuations lead the BC fluxes changed from 0.05 to 0.3 g C/m^2^.yr. While, after 1200 cal yr BP, the BC fluxes in two profiles were higher than 0.3 g C/m^2^.yr, and the BC fluxes changed from 0.3 to 0.9 g C/m^2^.yr. Because human activities increased and more BC emission from anthropogenic sources, the increasing trend of BC fluxes in two profiles were more clearly during the last 1200 years than before. Thus, anthropogenic sources may be a major component of BC sources that have affected BC deposition fluxes over the last 1200 years.

Background trends of BC fluxes, obtained by Charanalysis 1.1, reflect the characteristics of regional BC deposition fluxes^37^. Unlike trends in BC fluxes in the two profiles, background values can be easily removed from the local distribution to study the relationship between regional BC deposition and its contributing factors^38^,^39^. Background trends obtained from the two profiles were similar (Fig. 3b) and have a close relationship with population trends in HLJ Province. Compared with BC background fluxes in the HXZ profile, the trend in the DFH profile fit more closely with the trend of the historical population in HLJ Province, especially in the fluctuation trend that appeared around approximately 1000 cal yr BP. For the DFH profile, the proportion of anthropogenic sources that affect BC deposition fluxes was much higher than that of natural sources. Because of its greater distances from human habitation, fewer anthropogenic factors have disturbed the sedimentary process of the HXZ profile. With the climatic fluctuations of the Holocene epoch, fluxes in BC deposition in the HXZ profile more obviously varies relative to those in the DFH profile. The historical trend of BC fluxes in the HXZ profile show that the BC fluxes decreased during periods of cool climate and that the fluxes were lower than that during other periods adjacent to these cool periods. Overall, BC deposition fluxes were affected by human activities and climate change, while human activities were the major factors that have influenced BC fluxes in both profiles during the last 1200 years.

### Historical combustion characteristics revealed by different PAH ratios

**Figure 4:**
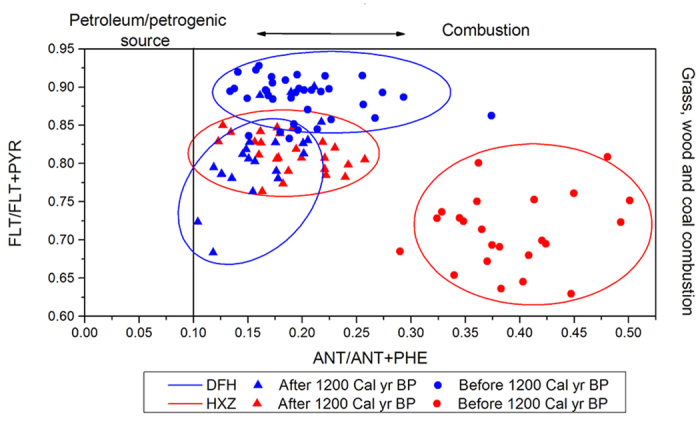
Cross plot of PAHs diagnostic ratios (FLT/(FLT + PYR) vs. ANT/(ANT + PHE)) in the two profiles (PHE: phenanthrene; ANT: anthracene; FLT: fluoranthene; PYR: pyrene). Boundaries for source assignments of PAHs were based on Yunker *et al*.^27^.

The ratios of different PAHs were useful for diagnosing the sources of PAHs^40^,^41^,^42^. Among the eight types of PAHs with low aromaticity, the ratio of FLT/(FLT + PYR) and ANT/(ANT + PHE) could be used to determine combustion and petroleum/petrogenic sources^27^,^31^. The results obtained from the ratio of PAHs in the two profiles are shown in Fig. 4. During the late Holocene, the ratios of FLT/(FLT + PYR) and ANT/(ANT + PHE) in the two profiles were both higher than 0.6 and 0.1, respectively, which indicated that historical PAHs were primarily caused by the burning of biomass (i.e., grass, coal and wood combustion)^31^.

Although the sources of combustion in the Sanjiang Plain during the late Holocene epoch primarily consisted of biomass burning, the diagnostic ratios of PAHs (i.e., FLT/(FLT + PYR) vs. ANT/(ANT + PHE)) were different in different periods. The different periods caused the diagnostic ratios of PAHs to be different at approximately 1200 cal yr BP. Before this period, the DFH profile had a higher FLT/(FLT + PYR) ratio and a lower ANT/(ANT + PHE) ratio compared with the HXZ profile. After 1200 cal yr BP, the ratio of ANT/(ANT + PHE) in the HXZ profile declined, while the ratio of FLT/(FLT + PYR) increased. Additionally, the ratio of FLT/(FLT + PYR) in the DFH profile decreased during that period. These variations caused the diagnostic ratio of PAHs in the two profiles to be similar after 1200 cal yr BP. Variation in PAH diagnostic ratios in the two profiles indicated the main types of combustion sources. Major contributions to PAHs were natural sources (e.g., wildfire), while different vegetation communities were responsible for the difference in the ratio of PAHs between the DFH and HXZ profiles. With the gradual effect of the Han farming culture on Northeast China during the Sui and Tang dynasties, population growth and wars caused the PAHs produced by human activities and affected the results of diagnostic ratios. Similar lifestyles around the two sampling sites may be the major reason for the similar diagnostic ratios of PAHs after 1200 cal yr BP.

BC, produced by incomplete combustion, was co-emitted with PAHs by combustion sources^26^. The types of combustion sources identified by the diagnostic ratios of PAHs were sources of BC during the late Holocene. BC was primarily produced by biomass burning (e.g., wildfire and residential combustion). Major sources of combustion products transitioned from natural to anthropogenic sources at approximately 1200 cal yr BP. Before 1200 cal yr BP, the sources of BC were primarily from vegetation wildfires and were affected by the alteration of climate temperature in the two profiles. Variation in source types can be used to explain why BC background deposition fluxes decreased during the periods of cool climate before 1200 cal yr BP (e.g., from 2200 cal yr BP to 1800 cal yr BP) and weakly influenced fluxes after 1200 cal yr BP (e.g., 800 cal yr BP). After 1200 cal yr BP, population growth became a major factor affecting historical BC deposition fluxes.

### Dating the beginning period of intensive anthropogenic influence

**Figure 5:**
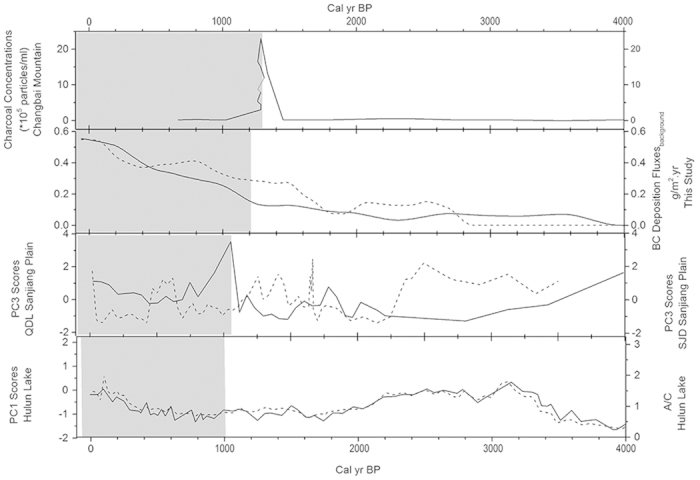
Historical anthropogenic influences on deposition records in different sites in Northeast China (from South to North). (**a**) Jinchuan peat, Changbai Mountain, obtained by charcoal analysis^43^. (**b**) HXZ (solid line) and DFH (dash line) wetland, Sanjiang Plain, obtained by background values of BC deposition fluxes. (**c**) QDL (solid line) and SJD (dash line) peat, Sanjiang Plain, obtained by the principal component analysis of geochemical analysis (PC3 scores)^7^. (**d**) Hulun Lake, northeastern Inner Mongolia, obtained by the principal component analysis of pollen analysis (PC1 scores, solid line) and the ratio of *Artemisia* to *Chenopodiaceae* (A/C, dash line)^44^. The gray region means human activities began to influence historical deposition records.

**Figure 6:**
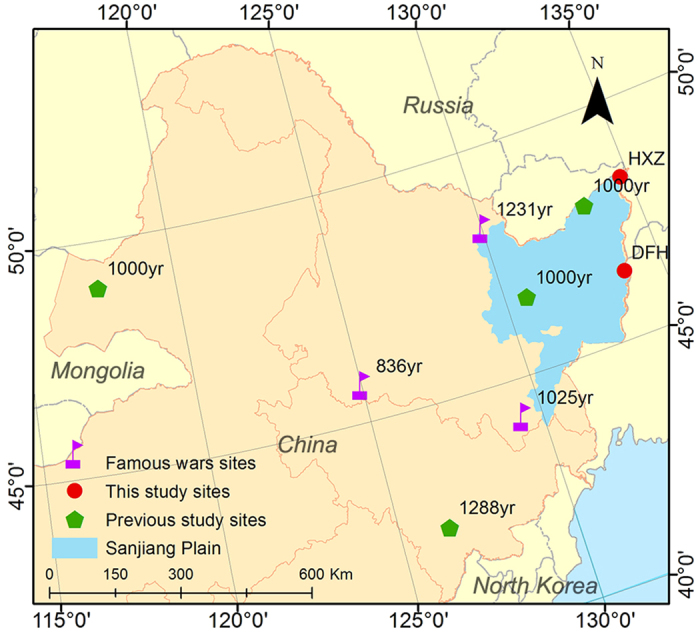
Location of the DFH (N 46.4°, E 133.8°) and the HXZ (N 48.3°, E 134.8°) profiles (red, circle) in the Sanjiang Plain, Northeast China. Previous studies sites (green, pentagon) around the Sanjiang Plain and the label for each site where the beginning of intensive anthropogenic influences on the ecosystem (cal yr BP) was conducted^7^,^43^,^44^ and prominent wars sites (purple, flag) with dates (cal yr BP) in Heilongjiang history^45^. (BP: before present, AD 1950). The figure was created using the results of remote sensing interpretation and was generated by Chuanyu Gao using ArcMap 10.0.

Several previous studies have examined the influence of historical human activities on sedimentary records and have identified the periods when intensive anthropogenic influences around the Sanjiang Plain through peatland or lake sedimentary archives began (Fig. 5). Charcoal records in the Jinchuan peatland (south of the Sanjiang Plain) show that the Han farming culture influenced northeastern China and caused fire events at approximately 1288 cal yr BP^43^. Trace element records in two peat profiles on the Sanjiang Plain suggest that human activities have increased deposition fluxes approximately 1000 cal yr BP^7^. Pollen analyses of Hulun lake sediments (west of the Sanjiang Plain) suggest that human activities significantly influenced this region and that pollen records changed after 1000 cal yr BP^44^. As discussed in previous sections, historical black carbon deposition fluxes increased after 1200 cal yr BP. Additionally, the diagnostic ratio of PAHs changed at approximately 1200 cal yr BP, and the diagnostic ratios of PAHs were similar after 1200 cal yr BP across different profiles. The increasing trend in combustion products (i.e., black carbon fluxes) and the change in source characteristics (i.e., the diagnostic ratios of PAHs) may indicate that wetland ecosystems in the Sanjiang Plain were extensively influenced by human activities at approximately 1200 cal yr BP. The combination of our research results with previous studies in different sedimentary settings near the Sanjiang Plain indicates that human activities began to influence the ecosystem between 1200 and 1000 cal yr BP (Fig. 6).

Sun and Li^45^ note that there were three notable wars in the history (Before AD 1200) of Heilongjiang Province (Fig. 6). The earliest recorded war in the Heilongjiang Province occurred in the northwestern Sanjiang Plain from AD 719 to 726 (1231-1224 cal yr BP). Two other prominent wars in Heilongjiang Province occurred in AD 925 and AD 1114. Both wars were followed by a change in the ethnicity of the rulers. These wars and the changing ethnic polices may be the major reason for the violent fluctuation in the population of HLJ Province from 1200 to 800 cal yr BP)^4^. Because human activities were more intensive during the wars than during peaceful times, wartime can be considered a time when human activities became the major factor affecting the ecosystems. Before 1200 cal yr BP, the population of HLJ Province was less than 100,000^4^, and the emission of combustion products or other pollutants was generally low. Ecosystems without human influences can be considered as the baseline conditions for ecosystem conservation and management policies^3^. Overall, for the wetland ecosystem of the Sanjiang Plain, historical wetland conditions before 1200 cal yr BP were affected by few human activities. Thus, the conditions that characterized the wetland before this period can be considered as the baseline conditions necessary for wetland recovery.

## Method

### Site description and sampling

The HeiXiaZi (HXZ; Black Bear Island, in English) wetland is located in the eastern Sanjiang Plain in a delta region where the Wusuli and Heilong rivers converge. The Dongfanghong (DFH) wetland is located south of the Black Bear Island wetland, and its distance from the origin of the Wusuli River is different from that of the HXZ wetland (Fig. 1). The study area has a temperate humid monsoon climate with an annual temperature of approximately 2.7 °C and an annual mean precipitation of approximately 550 mm/year^46^. Detailed Information on the Sanjiang Plain can be found in Bao *et al*.^47^. Currently, HXZ is surrounded by *Deyeuxia angustifolia* communities, whereas *Carex lasiocarpa-Carex pseudocuraica* communities are present in DFH. Sediment samples (Fig. 1) were collected from exposed profiles dug into representative areas of the two wetlands in October 2010. The sampling sites were located at the DFH wetland (N46°25.18′, E133°48.41′; 100 cm) and the HXZ wetland (N48°19.85′, E134°44.76′; 100 cm). Collected profiles were sliced into 1-cm vertical sections using stainless steel knives and transported to the laboratory for further analysis. Samples were loosely disaggregated to facilitate air drying at 20 °C.

### Chronology

We obtained four radiocarbon dates for HXZ and two for DFH. Bulk sediments were prepared by following the protocol described in Zhou *et al*.^48^. Analyses were conducted using the acceleration mass spectrometer (AMS) facility in Xi’an. The ^14^C dates were converted to calibrated ages (cal. yr. BP) with CALIB version 7.0 (Table 1). Because the samples were collected in 2010, the top of the profiles were set to −60 BP. All ^14^C data were processed using the ‘Bacon’ piecewise linear accumulation model^49^ in the R environment^50^ to establish the age-depth model. This model quantifies the total chronological error and returns maximum age probabilities at 1-cm intervals^51^,^52^.

### Black carbon

We used the dichromate oxidation method developed by Song *et al*.^53^ to measure black carbon. First, inorganic carbon was removed by treating 1-g subsamples for 20 h in 10 ml of 1 mol/L HCl acid contained in plastic centrifuge bottles. After digestion, the concentrations were centrifuged, and the residue was added to a 10-mL mixture of HCl (3 mol/L) plus HF (22 mol/L) at a 1:2 volumetric ratio. The mixture was then centrifuged, and the residue was soaked in 1 mol/L HCl (10 ml) for 10 h. The second step involved the removal of NPOC (non-pyrogenic organic carbon) from the samples; 0.1 mol/L NaOH (30 ml, 12 h, twice) was used to remove humic acid, while kerogen was removed using a mixture of K_2_Cr_2_O_7_ (0.1 mol/L) and H_2_SO_4_ (2 mol/L) (60 h, the mixture that kept its yellow hue was kept). All steps were conducted in a 55°C bath^18^. Residual carbon was quantified as black carbon by Flash 2000 series at the Analysis and Test Center of Northeast Institute of Geography and Agroecology, Chinese Academy of Sciences. Standard samples with known carbon concentrations (IRMS certified reference: BN/132357) were used to calibrate the measurements and to monitor working conditions.

### PAHs

Sediment samples (20 g), combined with Na_2_SO_4_ (20 g), were extracted with 20 ml of hexane–acetone (1:1, v/v) at 20 °C under ultrasonication (10 min, twice). The extract was then concentrated (2 ml) with a termovap sample concentrator and solvent-exchanged with 5 ml of hexane in a rotary evaporator (1 ml). The separation was performed with a Na_2_SO_4_ (2 g)-silica gel (10 g)-Na_2_SO_4_ (3 g) column with a 10-mm diameter under 40 ml pentane solvent leaching. PAHs were eluted with 25 ml of hexane–dichloromethane (2:3, v/v) after aliphatic ethers had been removed with 20 ml of pentane. The elution was again concentrated, solvent-exchanged with 5 ml hexane comma and blown to 1 ml. Instrumental analysis was performed with a GC/MS system (QP5050A). The column oven temperature was initially held at 50 °C for 1 min, increased to 200 °C at 25 °C/min, held for 1 min, increased to 280 °C at 10 °C/min, and finally held for 30 min. The injector temperature was kept at 320 °C, and the ionization energy was 70 eV with highly pure helium as the carrier gas (constant flow rate of 1.5 ml/min). The SEPA Institute standard samples of 16 priority PAH standard mixtures were adopted as external standards for quantitative analysis. Because the concentrations of PAHs with several rings was less than the limit of detection in some layers, we choose eight types of PAHs with a low number rings that could be detected in all layers for this study.

### Background values of BC fluxes

The background deposition fluxes of BC were determined using CharAnalysis version 1.1^54^. The results of smoothing can be considered to represent background values and have been used to indicate regional fire events to reconstruct fire history through charcoal records^35^. Similar to charcoal records, BC fluxes are affected by both local and regional emissions and can be analyzed using similar methods^55^. The trend of regional BC deposition over the last 5000 years was obtained from the background value of BC deposition fluxes in the two profiles. The appropriate reciprocal of dry bulk density was considered to be the volume of the samples. Moving modes with 10-year interpolations were used to estimate background values (low-frequency) of BC fluxes.

## Additional Information

**How to cite this article**: Cong, J. *et al*. Dating the period when intensive anthropogenic activity began to influence the Sanjiang Plain, Northeast China. *Sci. Rep*. **6**, 22153; doi: 10.1038/srep22153 (2016).

## Figures and Tables

**Table 1:** Radiocarbon ages with calibrated years.

laboratory code	Sub.code	core	depth (cm)	^14^C yr BP	Error ( ±1σ)	cal yr BP	2σ range cal yr BP
XA7520	H-1-30	HXZ	29–30	1625	28	1521	1472–1570
XA7525	H-1-51	HXZ	50–51	3508	27	3777	3698–3856
XA7526	H-1-74	HXZ	73–74	5070	28	5825	5746–5904
XA7527	H-1-100	HXZ	99–100	8062	32	9000	8966-9034
XA7556	D-51	DFH	50–51	2582	30	2732	2700–2764
XA7557	D-99	DFH	98–99	4434	32	5009	4947–5071
